# Intention for Screening Colonoscopy among Previous Non-Participants: Results of a Representative Cross-Sectional Study in Germany

**DOI:** 10.3390/ijerph18084160

**Published:** 2021-04-14

**Authors:** Anne Starker, Franziska Prütz, Susanne Jordan

**Affiliations:** 1 Robert Koch Institute, Department of Epidemiology and Health Monitoring, General-Pape-Str. 62-66, 12101 Berlin, Germany; pruetzf@rki.de (F.P.); jordans@rki.de (S.J.); 2 Institute for Public Health, Charité–Universitätsmedizin Berlin, Charitéplatz 1, 10117 Berlin, Germany

**Keywords:** colonoscopy, colorectal cancer screening, non-participation, intention, secondary prevention, health literacy, utilisation, gender

## Abstract

Early detection of colorectal cancer has the potential to reduce mortality at population level. Colonoscopy is the preferred modality for colon cancer screening and prevention, but attendance rates are low. To exploit colonoscopy’s preventive potential, it is necessary to identify the factors influencing uptake, especially among previous non-participants. This analysis of cross-sectional data involved 936 non-participants in screening colonoscopy aged 55 years or older in Germany. Differences between non-participants with and without future participation intentions were investigated in terms of socio-demographic factors, health status, attitudes and beliefs, and medical counselling. Logistic regression models were fitted to estimate associations between intention to participate and selected factors. Intention to participate was lower among women than among men. For both genders, intention to participate was positively associated with younger age. For women, higher socioeconomic status and counselling were positively associated with intention to participate. Men showed a positive association with favouring joint decision-making. The results draw attention to starting points for improving acceptance of and participation in screening colonoscopy. This includes good medical counselling and successful physician–patient communication, for which the information and communication skills of both medical professions and the general public should be strengthened. Gender differences should be considered.

## 1. Introduction

Colorectal cancer (CRC) is currently one of the three most common cancers in Germany. In 2016, about 25,990 women and 32,300 men were diagnosed with CRC [1]. This means that 1 in 20 women and 1 in 17 men will be diagnosed with the disease in the course of their lives. In Germany, mortality rates of patients with CRC have decreased significantly over the last decade. Nevertheless, 11,391 women and 13,411 men died with this diagnosis in 2016 [1]. Early detection and treatment of CRC are interventions that have been shown to reduce mortality at population level [2,3,4,5]. Colonoscopy is the preferred modality for both colon cancer screening and colon cancer prevention, because during colonoscopy the colon is examined for potentially cancerous tissue, as well as proliferation of the intestinal mucosa (intestinal polyps) which can usually be removed during the examination. The patient’s prognosis for CRC depends in particular on the stage of the tumour, with better outcomes associated with earlier stages. Within 10 years after the introduction of screening colonoscopy in Germany, the incidence of colorectal cancer has decreased in the age groups 55 and older, which is considered a success of screening colonoscopy [3]. In Germany, CRC screening (CRCS) is regulated by law, and the costs of these examinations are covered by all statutory health insurers, which insure approximately 86% of the population. Individual participation in CRCs is optional. In Germany, two examination methods are used: insured persons aged 50 years and older can choose between faecal occult blood testing (FOBT) and screening colonoscopy. The tests are offered at scheduled intervals depending on age and gender, and there is a choice between the two alternatives [6]. If the result of the FOBT is positive, this test is followed by diagnostic colonoscopy. Since there are screening options, medical counselling is provided by all physicians who perform CRCS examinations. As part of this counselling, the following aspects should be explained: frequency and disease pattern of CRC; the goal and concept of screening examinations; the examinations’ sensitivity, specificity, efficiency (reducing mortality from CRC) and possible disadvantages (burdens and medical risks); and the way forward in case of a positive finding [7].

Data from the statutory health insurance system show that CRCS uptake rates are low in Germany. For example, the uptake of FOBT was 19.8% of eligible women and 15.7% of eligible men from 2017 to 2018. The cumulative uptake of screening colonoscopy from 2009 to 2018 was 15.1% among eligible women and 13.8% among eligible men [8]. Low uptake rates for FOBT and colonoscopy have also been reported in other European countries [9,10].

Screening colonoscopy offers the potential for both primary and secondary prevention. To exploit this preventive potential, it is necessary to identify the factors that influence uptake, especially among those who have not yet participated. Socio-demographic factors, especially age, gender and social status [11,12,13]; indicators of health [14]; and aspects of physician–patient communication, especially physician recommendation, are considered to influence participation [15,16,17]. Attitudes and beliefs—both decision-making preferences in general and those that are specific to the use of a screening examination (e.g., whether shared decision-making is favoured)—are also assumed to have an impact [18,19,20]. In addition, access to the healthcare system and the reimbursement of examination costs may also be important [21]. However, most of studies on this topic have been conducted in the United States, Great Britain or the Netherlands and, therefore, cannot be easily transferred to the German healthcare system context or the doctor-patient situation in Germany.

Non-participants in CRCS and their intentions to have a colonoscopy for CRCS have not yet been investigated in a representative study in Germany. Data are also lacking on the association of socio-demographic factors, health status, health-related attitudes and beliefs, and medical counselling with intention to have a colonoscopy.

The aim of the present study was therefore to examine the following research questions:

1. Among the group of previous non-participants in CRCS, do those who intend to have a screening colonoscopy differ from those who do not?
2. What are the determinants of intention to have a screening colonoscopy among non-participants?
3. Is intention to have a screening colonoscopy associated with physician counselling?

## 2. Materials and Methods

### 2.1. Study Design and Study Population

The present analyses are based on data from the study ‘KomPaS: Survey on Communication and Patient Safety’, a representative cross-sectional telephone survey of the German-speaking residential population aged 18 years and over living in a private household in Germany, conducted by the Robert Koch Institute. In the survey period (May–September 2017), a total of 5053 people were interviewed. The sample was drawn using the dual-frame method (ratio of 60% landline numbers to 40% mobile numbers) from the ADM Telephone Survey System provided by the ADM Arbeitskreis Deutscher Markt- und Sozialforschungsinstitute e.V. (ADM Working Group of German Market and Social Research Institutes). The response rate according to criteria of the American Association for Public Opinion Research (AAPOR Outcome Rate Calculator; Dual-Frame RDD phone; Version 4.0, May, 2016) was 17.2%. The aim of the study was to describe different areas of information behaviour, decision-making behaviour and communication behaviour of patients, as well as the physician–patient relationship, from a population perspective. The questionnaire developed for the KomPaS study was tested by a qualitative and quantitative pre-test. For the qualitative testing of the questionnaire, we cooperated with an external research institute specialized in questionnaire testing. The detailed study procedure and methodology have been published elsewhere [22,23].

One part of the study focused on the early detection of CRC. For this purpose, 2901 people aged 55 years or older were interviewed because people of this age are entitled to a colonoscopy for the early detection of CRC [24]. The items used in the questionnaire were based on survey instruments that had previously been used in other studies [25,26,27,28]. The study participants were asked whether they had ever had a colonoscopy (answer options: yes, no). All individuals who provided a negative answer to this question were classified as previous non-participants—the study population of the present analyses (*n* = 936).

### 2.2. Indicators

Intention to participate in a screening colonoscopy, the outcome measure in this study, was assessed with the following question: ‘Do you intend to have a colonoscopy for early cancer detection in the future?’ (yes, no).

The following determinants were examined as independent variables: in addition to age and gender, information on education, occupational status and income was collected to determine socioeconomic status (SES; low, medium or high) [29].

Several questions were asked to assess different aspects of health: self-perceived health (‘How is your health in general? Is it very good, good, fair, bad or very bad?’); long-standing health problems (‘Do you have any longstanding illnesses or health problems?’ (yes, no) and limitations in activities because of health problems (‘Are you limited because of a health problem in activities people usually do? Would you say you are severely limited, limited but not severely or not limited at all?’).

Health awareness was assessed by asking ‘How strongly do you generally pay attention to your health?’ (very strong, strong, moderate, less strong, not at all), and health-related locus of control was assessed by asking ‘How much can you do yourself to maintain or improve your health?’ (nothing, little, some, much, very much). Treatment decision-making preferences were measured by asking ‘Which of the following statements would you most agree with? My doctor should keep me informed, but in general he should decide how best to treat me; My doctor should discuss the different treatment options with me, and we will then come to a joint decision; and My doctor should explain the different treatment options and the pros and cons, and then I will decide what to do’. We categorized the answers as ‘physician’s decision favoured’, ‘joint decision making favoured’ or ‘own decision making favoured’.

The study participants were asked to recall previously conducted physician counselling when responding to the following question: ‘Has a doctor ever advised you about colon cancer and the existing possibilities for early detection (e.g., stool test or colonoscopy)?’ (yes, no).

### 2.3. Statistical Analyses

The analyses used data from 936 people aged 55 years or older who had not yet had a colonoscopy. Relative frequencies and 95% confidence intervals (CIs) are reported. Wider CIs indicate greater statistical uncertainty in the results (Table 1).

Using Pearson’s χ^2^ test, differences between non-participants with and without the intention to participate in screening colonoscopy in the future were analysed for statistical significance with regard to the determinants described above. A significant group difference was assumed if the *p*-value calculated considering the weighting and the survey design was smaller than 0.05. Multivariate binary logistic regression was used to test for associations between intention to participate in screening colonoscopy in the future (the outcome variable) and the tested determinants. For this purpose, odds ratios (ORs) were calculated for intention to participate in screening colonoscopy in the future with 95% CIs and *p*-values. All analyses were conducted with Stata 15.1 [30] using survey procedures for complex samples, which allows weighting to be appropriately taken into account when calculating CIs and *p*-values. Weighting factors were used to correct for sample deviations from the population structure in terms of age, gender, education and place of residence (community type), referring to German Federal Statistical Office data for 2017 [23].

## 3. Results

### 3.1. Comparison of Non-Participants with and without Intention to Participate in Screening Colonoscopy in the Future

Group differences in socio-demographic factors, health status, attitudes and beliefs, and physician counselling were examined (Table 2). Significantly fewer women than men expressed an intention to participate in screening colonoscopy (*p* = 0.0001). Regarding the other examined socioeconomic factors, intention to participate in screening colonoscopy decreased significantly with age for both genders. Men with high SES expressed the lowest intention; among women, intention was lowest among those with low SES (differences not significant). For both men and women, there were no significant differences between individuals who intended to participate in screening colonoscopy and those who did not in terms of health status indicators. One attitude and belief determinant had a significant effect only among men: the lowest level of intention to participate in screening colonoscopy was expressed by those who favoured physician decision making. For physician counselling, intention to participate in screening colonoscopy varied significantly only among women, who had significantly lower levels of intention to participate if they had not received counselling than if they had received counselling.

### 3.2. Associations between Intention to Participate in Screening Colonoscopy and the Examined Determinants

Because of group differences, binary logistic regression was used to test the associations between the determinants and intention to participate in screening colonoscopy in the future (Table 3). In addition to the raw ORs, we present the ORs from a model that adjusted for respondent’s age and SES to examine the extent to which independent associations existed between intention to participate in screening colonoscopy and the examined determinants.

Men and women aged 55–59 years or aged 60–64 years had increased odds of intention to participate, compared with those aged 65 years or older. Women with medium or low SES had lower odds of intending to participate in screening colonoscopy compared with women with high SES. Men who favoured joint decision making had a significantly higher odds of intending to participate compared with those who favoured a physician’s decision. This effect increased after adjusting for age and SES. Among women, those who had received physician counselling had a significantly higher odds of intending to participate compared with those who had not received such counselling. Here, too, the effect intensified after adjustment, indicating a strong independent association.

## 4. Discussion

Summarised this is the first representative study on non-participants in CRCS and their intentions to have a colonoscopy for CRCS in future in Germany. We, first, generated data on the association of socio-demographic factors, health status, health-related attitudes and beliefs, and medical counselling with intention to have a colonoscopy. The main findings are: (1) The intention to participate is lower for women than for men and for both genders, intention to participate was positively associated with younger age; and (2) determinants of intention to have a screening colonoscopy among non-participants are for women higher socioeconomic status and counselling and for men favouring joint decision making.

Our results show age-related differences in the intention to participate in screening colonoscopy: people aged under 65 years express a higher level of intention to participate than do older individuals. This finding was also reported in a previous study [31]. The data on CRCS colonoscopy attendance in Germany show lower participation for the 55–59 years age group compared with older age groups [9,32]. Thus, previous non-participants aged under 65 years seem to intend to participate but probably in the distant future (i.e., at older ages). This finding may also be related to people realising that the probability of CRC increases with age; however, this understanding could also increase the fear of being diagnosed with CRC, which may reduce willingness to participate [33]. Furthermore, medical reasons that increase with age, maybe contrary to participation intentions such as a non-functioning bowel or a terminal illness [34]. We did not investigate these aspects in our study.

We found, that intention to participate in screening colonoscopy is associated with SES for women but not for men. This finding is in line with a previous report of little difference in men’s CRCS (FOBT and colonoscopy) attendance by SES [35]. The results for women are also consistent with previous studies indicating that women with higher SES are more likely to use preventive services [36] and have a higher level of intention to participate in CRCS [37]. The association between SES and intention to participate is explained by people with higher SES being better informed, having less fear of the examination and being more likely to consider cancer screening useful [38].

Attitude towards decision making is considered an influencing factor for participation in CRCS [20]; this idea is confirmed by the present results for men. A US study conducted by Messina et al. [20] showed that previous non-participation in CRCS was associated with a lower likelihood of involving a physician in CRCS decisions; however, the authors acknowledged that people who prefer to make screening decisions on their own are more likely to have negative rather than positive attitudes towards screening, compared with those with other decision-making preferences, and that attitudes towards screening are probably more important than a preference for a particular type of decision making. In our survey, treatment decision-making preferences were assessed in general terms not specific to CRCS, so the results are not directly comparable to previous work. Because of the different screening options for CRCS in Germany and the counselling provided for this reason, the association between the intention to participate and favouring a joint treatment decision is understandable. This finding is in line with the results of previous studies on the patient–doctor relationship in Germany, which show that joint decision making is favoured in general and in the context of early cancer detection [28,39]. Joint or shared decision making is positively associated with making informed decisions, including regarding cancer screening [40]. A decision is considered informed if it is based on sufficient knowledge about the advantages and disadvantages of the examination, in accordance with personal attitudes, which are then reflected in the decision for participation or non-participation [41].

In our results we found an association between intention to participate in screening colonoscopy and previous physician counselling, but only for women. A good physician–patient relationship is attributed to a positive influence on the course of disease and the success of treatment. A precondition for a good relationship is successful communication. Counselling, as a communication situation in the physician–patient relationship, is of major importance for the communication of health information. Despite the availability of health information from several sources, for most patients, the physician is the main point of contact for health-related questions [42]. Successful physician–patient communication depends on networking and co-operation between all parties involved [43]. Our findings are consistent with studies that have shown that the physician’s recommendation is a major contributing factor to whether CRCS examinations are taken up [39,44,45]. Studies on the content of physician–patient discussions on CRCS emphasize the importance of understanding which aspects of the discussion content facilitate an informed and value-compliant decision that could ultimately increase the uptake of CRCS [45,46]. We did not have information on the type or content of physician counselling in our study. Further research is needed in this area.

Our results indicate a difference in intention to participate in colonoscopy for CRCS between women and men, with more men than women expressing an intention to participate. A previous study also reported this result [47], but other studies have found no gender difference in intention to participate [48]. One explanation for the gender difference could be gender-specific barriers. In a qualitative study [49], women most frequently mentioned the intrusive examination method, the fear of perforation and embarrassment; for men, the most frequently cited reasons not to participate were avoiding the examination, not seeing any benefit in screening and the male role position being violated by the examination. Interestingly, there is little difference between women and men in Germany in the uptake of colonoscopy for the early detection of CRC [9,32], in contrast to the other statutory cancer screening examinations, which show a clear gender difference in favour of women [36]. The examined determinants also show gender-specific differences, supporting calls to make the offer of preventive services more gender sensitive. Furthermore, these findings make clear the necessity of adapting CRCS information and offers to the specific needs of women and men, especially considering their different morbidity and mortality rates [50,51].

The findings of our study draw attention to starting points for improving acceptance of and participation in screening colonoscopy, which are, however, associated with various challenges. Physicians must integrate counselling time into their daily medical routines, but there is a lack of incentives to do this in Germany because of low reimbursement [52]. Physicians are also responsible for providing counselling to women and men of different ages with different risk profiles, different levels of knowledge regarding cancer screening and different attitudes towards cancer screening and treatment decisions. Physicians need to be aware of these differences and tailor their efforts to the needs of their patients [53]. In their role as counsellors, doctors should develop their communication skills, and they need evidence-based and quality-assured information for their counselling. Additionally, medical students should already be prepared for later communication with patients during their studies. This has begun to be implemented in Germany [54]. However, not only those in the medical professions, but also the general population should be strengthened with regard to their information and communication competence. In Germany, attempts are being made to strengthen these aspects through specific measures to promote health literacy (e.g., through the Alliance of Central Actors in Health Care and the National Health Literacy Action Plan [55] as well as the National Cancer Plan [56]), with the aim of promoting both personal and organisational health literacy [57]. In Germany, there has been a health policy reorientation of early cancer detection since the beginning of the first decade of the 21st century, including the introduction of new legal regulations [58]. In accordance with European recommendations [59], an organised and quality-assured screening programme with an invitation and information system has been implemented in Germany since 2018 [6].

The KomPaS study was a snapshot in the time before the start of the organised and quality-assured screening programme. In order to evaluate the resulting possible improvement in the acceptance of colorectal cancer screening, a study on the intention to participate could be conducted again at a certain time interval. For future studies on this topic, we also propose to consider psychological factors such as fear of the complications of colonoscopy. In this way, existing gaps in the provision of information and in medical counselling could be identified.

The present study has several limitations that should be taken into account when generalising the results. Some limitations result from only certain determinants of intention to participate in screening colonoscopy being measured. Respondents may also remember the corresponding answer categories inaccurately (recall bias) or give socially desirable answers (social desirability bias), especially for intention to participate. Additionally, we did not have information on whether the participants had previously undergone FOBT for CRCS, their motivation for this type of examination or whether they reject cancer screening in general. We also did not have any information about the type or content of physician counselling, so we could not assess whether the benefits and risks of screening colonoscopy were explained in accordance with the guidelines or whether the doctors actively recommend the examination. Because of the study’s cross-sectional design, it was not possible form conclusions about the causality of the results. It is also known that telephone interviews are more susceptible to socially desirable response behaviour, compared with face-to-face interviews [60]. In addition, there is the possibility of bias caused by selective non-participation (selection bias). People who participate in a health survey can be assumed to have relatively high health awareness and therefore to differ from the general population in terms of non-participation in CRCS. Furthermore, certain population groups (e.g., individuals with a migration background or without sufficient knowledge of German) may be underrepresented in the sample because the interviews were conducted in German.

Possible selection effects were addressed by weighting, so the observed results are generalisable for Germany, which is a strength of the KomPaS study. In the present study, non-participants were examined using a representative population sample, and, because of the large sample size, we were able to conduct separate analyses for men and women. Our study is therefore a good supplement to analyses of statutory health insurance data and offers a starting point for the improvement and further development of CRCS.

## 5. Conclusions

The present study examined non-participants and their intention to participate in screening colonoscopy in the future. A preference for joint decision making and having received of physician counselling were found to be relevant factors. These findings draw attention to several potential starting points for improving screening colonoscopy acceptance and attendance. Strengthening the information and communication skills for doctors as well as for the general population is an essential aspect of this. Personal invitations with detailed information about the benefits and possible risks of the examinations should make it easier for individuals to decide whether or not to take advantage of these offers. These changes may also support the formation of intentions to participate among previous non-participants, in the sense of enabling them to make an informed decision.

To reduce barriers to intention to participate, age- and gender-specific needs, as well as attitudes and beliefs, should be taken more into account. However, further knowledge is needed, for example, to better explain gender differences and to evaluate the content and quality of medical counselling.

## Figures and Tables

**Table 1:** Characteristics of the study population aged 55 years or older.

		*n* (*Unweighted*)	% (Weighted)	95% CI
***Socio-demographic factors***				
Gender	Male	409	46.8	42.8–50.8
	Female	527	53.2	49.2–57.2
Age	55–59 years	206	26.2	22.9–29.8
	60–64 years	200	21.8	18.7–25.3
	≥65 years	476	52.0	48.0–55.9
Socioeconomic status	Low	106	20.5	17.0–24.5
	Medium	473	58.3	54.3–62.2
	High	336	21.2	18.7–24.0
	Missing	21		
***Health status***				
Self-perceived general health	Very good	147	13.1	10.8–15.7
	Good	464	47.2	43.2–51.2
	Fair	235	29.1	25.5–32.9
	Bad	75	8.9	6.8–11.6
	Very bad	14	1.7	1.0–3.1
	Missing	1		
Long-standing health problem	Yes	468	50.3	46.3–54.3
	No	466	49.7	54.7–53.7
	Missing	2		
Limitation in activities because of health problems	Severely limited	130	16.1	13.2–19.4
	Limited but not severely	257	31.3	27.6–35.3
	Not Limited	548	52.6	48.6–56.6
	Missing	1		
***Attitudes and beliefs***				
Health awareness	Not at all	18	2.3	1.3–3.9
	Less strong	47	5.0	3.6–6.9
	Moderate	353	38.7	34.9–42.6
	Strong	378	40.8	36.9–44.8
	Very strong	140	13.3	10.8–16.2
Health-related locus of control	Nothing	2	0.2	0.0–0.7
	Little	19	2.5	1.4–4.5
	Some	239	28.0	34.5–31.7
	Much	325	34.0	30.3–37.9
	Very much	348	35.3	31.6–39.2
	Missing	3		
Treatment decision-making preferences	Physician’s decision favoured	110	14.1	11.3–17.5
	Joint decision-making favoured	516	54.1	50.0–58.1
	Own decision-making favoured	275	31.8	28.1–35.8
	Missing	35		
***Counselling***				
Counselling on CRC and screening options	Yes	582	60.6	56.6–64.4
	No	353	39.4	35.6–43.4
	Missing	1		

Abbreviations: CI: confidence interval, CRC: colorectal cancer.

**Table 2:** Intention to participate in screening colonoscopy by the examined determinants (relative frequencies expressed as percentages).

	Men			Women		
	%	95% CI	*p*-Value	%	95% CI	*p*-Value
Total	44.3	38.1–50.6		28.8	24.2–33.9	
***Socio-demographic factors***						
**Age**						
55–59 years	68.2	56.9–77.7	0.0000	42.7	32.3–53.7	0.0000
60–64 years	49.7	35.7–63.8		39.1	28.9–50.2	
≥ 65 years	27.5	19.9–36.6		18.8	13.7–25.4	
**Socioeconomic status**						
Low	42.3	25.1–61.7	0.3856	25.4	16.0–37.9	0.0732
Medium	48.3	39.4–57.3		27.1	21.3–33.7	
High	37.7	30.0–46.1		42.2	33.0–52.0	
***Health status***						
**Self-perceived general health**						
Very good/good	44.6	36.9–52.5	0.9073	32.2	26.3–38.6	0.1113
Fair/bad/very bad	43.8	33.8–54.4		23.8	16.8–32.5	
**Long-standing health problem**						
Yes	41.1	32.4–50.4	0.2965	29.3	22.7–36.9	0.8853
No	47.8	39.2–56.6		28.6	22.5–35.6	
**Limitation in activities** **because of health problems**						
Severely limited/limited but not severely	38.1	29.2–47.9	0.0507	24.9	18.3–32.9	0.1524
Not limited	50.7	42.6–58.8		32.2	26.1–39.0	
***Attitudes and beliefs***						
**Health awareness**						
Moderate/less strong/not at all	43.8	35.4–52.6	0.8959	25.1	19.0–32.3	0.1667
Very strong/strong	44.6	35.9–53.8		31.9	25.4–39.1	
**Health-related locus of control**						
Nothing/little/some	38.1	27.0–50.6	0.2175	27.1	19.3–36.5	0.6049
Much/very much	47.1	39.8–51.1		29.9	24.4–36.1	
**Treatment decision-making preferences**						
Physician’s decision favoured	26.2	14.5–42.6	0.0182	33.4	18.8–52.1	0.4384
Joint decision making favoured	52.2	44.0–60.3		31.9	25.4–39.1	
Own decision making favoured	45.0	32.5–58.3		25.1	18.2–33.6	
***Counselling***						
**Counselling on CRC and screening options**						
Yes	48.8	40.6–56.2	0.1199	40.5	33.9–47.4	0.0000
No	37.7	27.7–48.8		10.9	6.8–17.0	

Abbreviations: CI: confidence interval, CRC: colorectal cancer.

**Table 3:** Odds ratios of intention to participate in screening colonoscopy by the examined determinants.

	Men				Women			
	OR (95% CI)	*p*-Value	Adjusted * OR (95% CI)	*p*-Value	OR (95% CI)	*p*-Value	Adjusted * OR (95% CI)	*p*-Value
**Age**								
55–59 years	5.68 (2.98–10.80)	0.000	-		3.21 (1.79–5.76)	0.000	-	
60–64 years	2.61 (1.28–5.33)	0.008	-		2.76 (1.53–5.00)	0.001	-	
≥65 years	1.00 (Ref.)		-		1.00 (Ref.)		-	
**Socioeconomic Status**								
Low	1.21 (0.52–2.85)	0.656	-		0.47 (0.23–0.94)	0.034	-	
Medium	1.54 (0.94–2.54)	0.087	-		0.51 (0.31–0.84)	0.009	-	
High	1.00 (Ref.)		-		1.00 (Ref.)		-	
**Treatment decision-making preferences**								
Physician’s decision favoured	1.00 (Ref.)		1.00 (Ref.)		1.00 (Ref.)		1.00 (Ref.)	
Joint decision making favoured	3.08 (1.37–6.92)	0.006	3.99 (1.58–10.09)	0.004	0.93 (0.40–2.15)	0.872	0.77 (0.33–1.82)	0.551
Own decision making favoured	2.31 (0.93–5.74)	0.071	2.73 (0.94–7.91)	0.064	0.67 (0.28–1.61)	0.369	0.63 (0.26–1.54)	0.307
**Counselling on CRC and screening options**								
No	1.00 (Ref.)		1.00 (Ref.)		1.00 (Ref.)		1.00 (Ref.)	
Yes	1.55 (0.89–2.69)	0.120	1.42 (0.77–2.62)	0.261	5.56 (3.10–10.03)	0.000	5.92 (3.20–10.95)	0.000

* Adjusted for age and socioeconomic status; Abbreviations: CI: confidence interval, CRC: colorectal cancer, OR: odds ratio, Ref.: reference category.

## Data Availability

The data presented in this study are available on request from the corresponding author. The data are not publicly available because informed consent from study participants did not cover public deposition of data. However, the minimal data set underlying the findings is archived in the ‘Health Monitoring’ Research Data Centre at the Robert Koch Institute (RKI) and can be accessed by all interested researchers. On-site access to the data set is possible at the Secure Data Center of the RKI’s ’Health Monitoring’ Research Data Centre. Requests should be submitted to the ’Health Monitoring’ Research Data Centre, Robert Koch Institute, Berlin, Germany (e-mail: fdz@rki.de).
