# Correction: Starker et al. Intention for Screening Colonoscopy among Previous Non-Participants: Results of a Representative Cross-Sectional Study in Germany. *Int. J. Environ. Res. Public Health* 2021, *18*, 4160

**DOI:** 10.3390/ijerph20105809

**Published:** 2023-05-12

**Authors:** Anne Starker, Franziska Prütz, Susanne Jordan

**Affiliations:** 1 Robert Koch Institute, Department of Epidemiology and Health Monitoring, General-Pape-Str. 62-66, 12101 Berlin, Germany; 2 Institute for Public Health, Charité–Universitätsmedizin Berlin, Charitéplatz 1, 10117 Berlin, Germany

## Additional Affiliation(s)

In the published publication [1], there was an error regarding the affiliation(s) for **Anne Starker**. In addition to **1**, the affiliation(s) should be **1,2**. The newly added affiliation 2 should be: “Institute for Public Health, Charité–Universitätsmedizin Berlin, Charitéplatz 1, 10117 Berlin, Germany”.

The authors apologize for any inconvenience caused and state that the scientific conclusions are unaffected. The original publication has also been updated.
